# *Bacillus coagulans* GBI-30, 6086 improves amino acid absorption from milk protein

**DOI:** 10.1186/s12986-020-00515-2

**Published:** 2020-10-23

**Authors:** Richard A. Stecker, Jessica M. Moon, Travis J. Russo, Kayla M. Ratliff, Petey W. Mumford, Ralf Jäger, Martin Purpura, Chad M. Kerksick

**Affiliations:** 1 Exercise and Performance Nutrition Laboratory, School of Health Sciences, Lindenwood University, St. Charles, MO 63301 USA; 2 Increnovo, LLC, Milwaukee, WI USA

**Keywords:** *Bacillus coagulans*, Probiotic, Amino acid, Protein, Absorption, Supplements

## Abstract

**Background:**

Probiotic *Bacillus coagulans* GBI-30, 6086 (BC30) has been shown to increase protein digestion in an in vitro model of the stomach and small intestine. Once active in the small intestine after germination, BC30 aids the digestion of carbohydrates and proteins. The extent to which BC30 administration may impact protein digestion and amino acid appearance in humans after protein ingestion is currently unknown. This study examined the impact of adding BC30 to a 25-g dose of milk protein concentrate on post-prandial changes in blood amino acids concentrations.

**Methods:**

14 males and 16 females (n = 30, 26.4 ± 6.5 years; 172.3 ± 10.8 cm; 78.2 ± 14.8 kg; 22.6 ± 7.2% fat) completed two supplementation protocols that each spanned two weeks separated by a washout period that lasted three weeks. Participants were instructed to track their dietary intake and ingest a daily 25-g dose of milk protein concentrate with (MPCBC30) or without (MPC) the addition of BC30. Body composition and demographics were assessed upon arrival to the laboratory. Upon ingestion of their final assigned supplemental dose, blood samples were taken at 0 (baseline), 30, 60, 90, 120, 180, and 240 min post-consumption and analyzed for amino acid concentrations.

**Results:**

Arginine (*p* = 0.03) and Isoleucine (*p* = 0.05) revealed greater area-under-the curve (AUC) in MPCBC30 group compared to MPC. In addition, Arginine (*p* = 0.02), Serine (*p* = 0.01), Ornithine (*p* = 0.02), Methionine (*p* = 0.04), Glutamic Acid (*p* = 0.01), Phenylalanine (*p* = 0.05), Isoleucine (*p* = 0.04), Tyrosine (*p* = 0.02), Essential Amino Acids (*p* = 0.02), and Total Amino Acids (*p* < 0.01) all revealed significantly greater concentration maximum (C_Max_) in MPCBC30 compared to MPC. Finally, time to reach C_Max_ (T_Max_) was significantly faster for Glutamine (*p* < 0.01), Citrulline (*p* < 0.01), Threonine (*p* = 0.04), Alanine (*p* = 0.02) in MPCBC30 when compared to MPC. Greater mean differences between groups for AUC and C_Max_ in women when compared to the mean differences in men were found for several amino acids.

**Conclusion:**

In concert with previous in vitro evidence of improved protein digestion and amino acid appearance, these results reveal that adding BC30 to protein sources such as milk protein concentrate can improve AUC, C_Max_, and faster T_Max_. Follow-up research should examine differences between gender and explore how aging can impact these outcomes. Retrospectively registered on June 11, 2020 at ClinicalTrials.gov as NCT04427020.

## Introduction

Probiotics are commonly defined as live microorganisms that, when administered in adequate amounts, confer a health benefit on the host [1]. Over 100 years ago, it was suggested that humans could change their microbiota while replacing harmful microbes with useful microbes [2]. Today, probiotics are known to impact and be related to a host of healthful benefits and outcomes including: modulation of the production of various bacterial species in the gut, bolster gut barrier function, and improvement in many properties of the human immune system [3]. Probiotics can also limit pathogen adhesion to host tissue, and modulate the production of different metabolites such as vitamins, short-chain fatty acids, and molecules that act as neurotransmitters involved in gut-brain communication [4]. Beyond benefiting physiological systems, probiotics have been shown to impact the absorption and production of key nutrients, including minerals, carbohydrates, protein, cholesterol, and various forms of digestive enzymes [5–7].

*Bacillus coagulans* GBI-30, 6086 (BC30) is a lactic acid producing, spore-forming bacterial species that has exhibited a variety of functions [8]. Secondary to its spore-forming ability, BC30 can survive harsh conditions of the gut and produces enzymes that ultimately aid in the digestion of both carbohydrates and proteins [9]. In this respect, previous studies that administered BC30 have reported improvements in gastrointestinal symptoms and side effects such as abdominal pain and bloating [10, 11]. In addition, BC30 promotes the production of short-chain fatty acids critical to maintain the health and vitality of the lining of the gut [12] while also exhibiting anti-inflammatory potential in several cell types found in the gut [13]. The observed improvements in inflammation and strengthening of the immune system [13, 14] are suggested to be mechanistic links to in vitro and animal work that has indicated the ability of BC30 to improve the absorption of amino acids into the bloodstream [8, 9] as well as improve protein digestion from both milk and plant proteins [15].

Currently, limited evidence is available highlighting the potential impact of adding BC30 to intact protein sources in human participants. Various sources of protein are available in the human diet with milk protein being a commonly consumed source. Bovine milk is comprised of approximately 80% casein and 20% whey protein and, as such, each of these protein sources exhibit divergent digestive kinetics. Due to these differences the appearance of amino acids are impacted as well as their ability to stimulate changes in protein metabolism [16, 17]. For these reasons, the need to examine the impact of adding BC30 to acute dosing of milk protein is evident to identify its ability to impact the absorption of amino acids after oral ingestion. Therefore, the purpose of this study is to assess the impact of adding BC30 to a standard dose of milk protein concentrate to determine the absorption of amino acids into the bloodstream.

## Methods

### Overview of research design

**Fig. 1:**
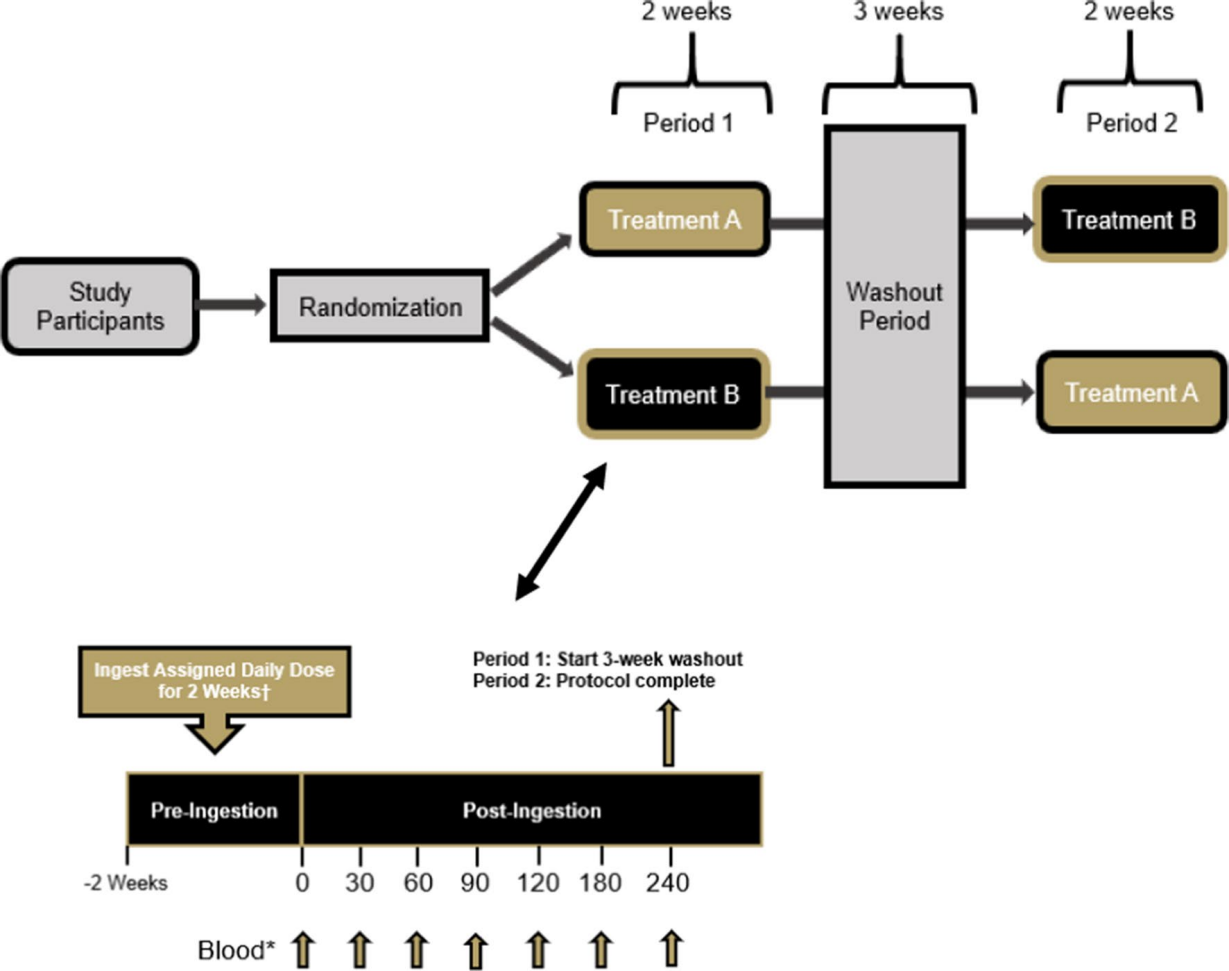
Overview of study design

**Fig. 2:**
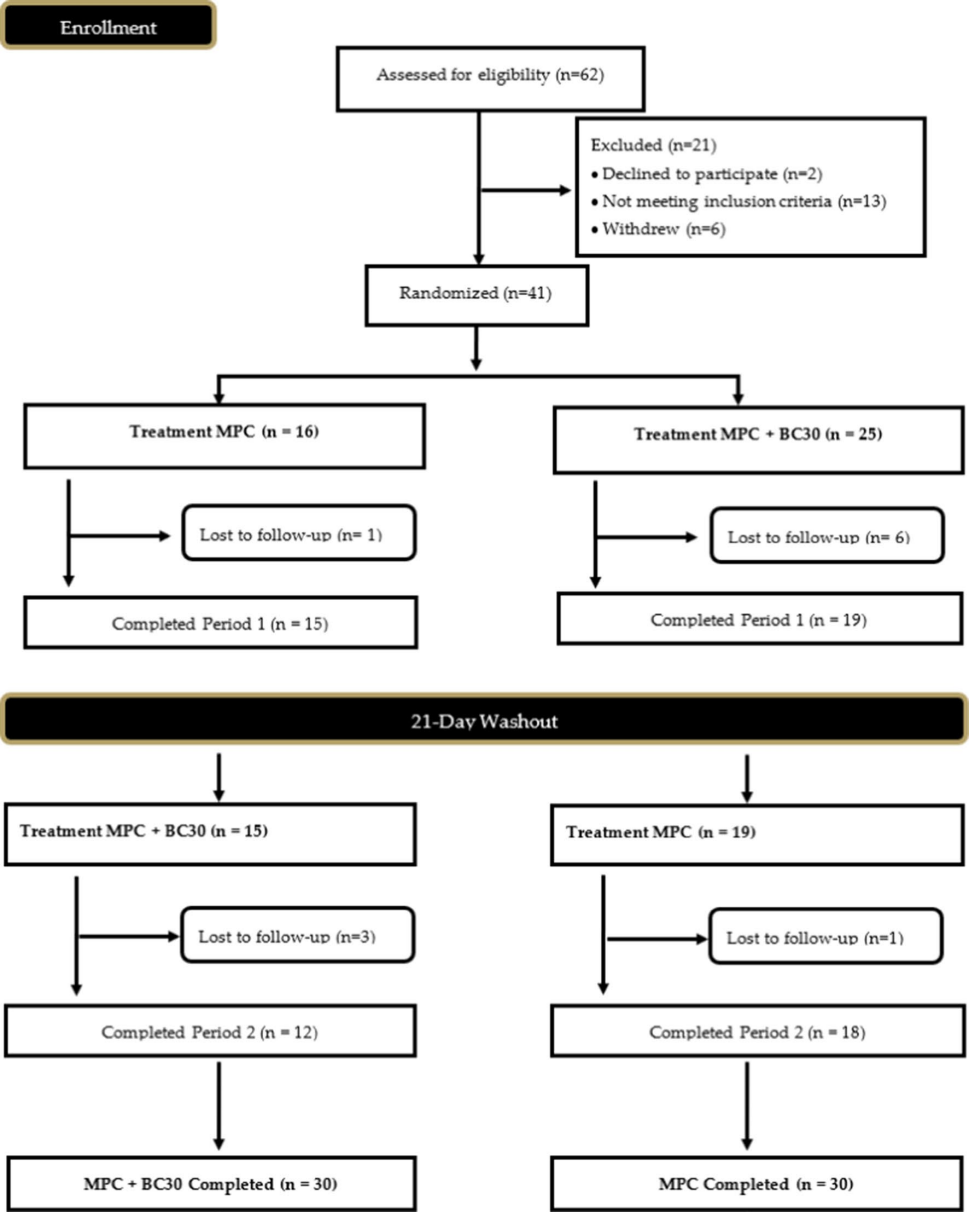
CONSORT diagram

The study was conducted using a randomized, double-blind, crossover study design. Healthy men (n = 14) and women (n = 16) between the ages of 18–55 years of age were recruited to participate in this study. Prior to beginning the study, all participants signed an IRB-approved informed consent document (Lindenwood University: IRB-20-15, approval date: 8/22/19) and completed a healthy history questionnaire to determine study eligibility. A priori sample size evaluation indicated that a sample size of 28–33 participants would be needed if an effect size of 0.5–0.55 was realized with an alpha (α) level of 0.05 and estimated power (1 − β) of 0.80. This study protocol and design was retrospectively registered on Clinicaltrials.gov on June 11, 2020 as NCT04427020 (https://clinicaltrials.gov/ct2/show/NCT04427020). Two supplementation protocols that each spanned two weeks were completed and separated with a washout period of three weeks (Fig. 1). For each study visit, all participants reported to the laboratory between 0600–1000 h after observing an 8–10-h fast. Prior to each study visit, participants were assigned in a randomized, double-blind, crossover fashion to ingest 13 daily 25-g doses of a milk protein concentrate or an identical dose of milk protein concentrate + BC30 (1 × 10^9^ colony forming units, CFU). To minimize any order effects from testing, participants were randomized using an online randomization software program (www.random.org). Upon arrival for each study visit, participants had their height, body mass, body composition and hemodynamics (resting heart rate and blood pressure) assessed. A series of venous blood collections were then collected. After collection of the first (0 min) blood sample, study participants ingested the 14th and final dose of their assigned supplement before having subsequent venous blood samples (~ 10 mL) collected 30, 60, 90, 120, 180, and 240 min after ingestion (Fig. 2). Participants were provided 200 mL of cold water to ingest after each blood collection. Upon processing, all blood samples were stored at − 80 °C. Prior to leaving, study participants were provided all doses of the alternative treatment to begin after observing a 3-week washout. After consuming 13 consecutive doses on the next assigned study treatment, participants returned to the laboratory for their remaining testing visit. All subsequent study visits were completed in an identical fashion.

### Study participants

Prior to participation, all recruited individuals provided signed informed consent using an IRB-approved consent form (Protocol IRB-20-15, approval date: 8/8/2019). In total, 14 males (26.9 ± 6.0 years; 180.9 ± 9.8 cm; 90.8 ± 9.1 kg; 18.3 ± 5.5% fat) and 16 females (25.9 ± 7.0 years; 165.0 ± 4.8 cm; 67.0 ± 8.2 kg; 26.4 ± 6.4% fat) successfully completed all study visits (see Table 1 for full participant characteristics). A Consolidation Standards of Reporting Trials (CONSORT) diagram was created to examine all study recruitment, randomization methods, and project completion and is provided in Fig. 2. Inclusion criteria included age (18–55 years), healthy and free of disease (as reported by the health screening questionnaire), and physically active (reported at least 30 min of moderate exercise three days a week). Any individual diagnosed with or being treated for cardiac, respiratory, circulatory, musculoskeletal, metabolic, obesity (defined as body mass index > 30 kg/m^2^ and body fat greater than 30%), immune, autoimmune, psychiatric, hematological, neurological, or endocrinological disorder or disease were not allowed to participate in the current study.

**Table 1:** Baseline age, gender, height (cm), weight (kg), body mass index, % fat, heart rate, systolic blood pressure, diastolic blood press, energy, carbohydrates, proteins, and fat intake

	Men (n = 14)		Women (n = 16)		Total (n = 30)	
	Mean	SD	Mean	SD	Mean	SD
Age	26.9	6.0	25.9	7.0	26.4	6.5
Height (cm)	180.6	9.8	165.0	4.8	172.3	10.8
Weight (kg)	90.9	9.1	67.0	8.2	78.2	14.8
Body mass index (kg/m^2^)	27.9	2.4	24.6	2.7	26.1	3.0
% fat	18.3	5.5	26.4	6.4	22.6	7.2
Heart rate (beats/min)	56.4	9.2	64.4	10.4	60.7	10.5
Systolic blood pressure (mm Hg)	124.9	8.8	109.3	9.6	116.6	12.1
Diastolic blood pressure (mm Hg)	66.6	7.3	68.2	8.2	67.4	7.7
Energy intake (kcal/day)	2747	1276	1693	604	2185	1098
Carbohydrate intake (g/day)	271	146	186	61	226	116
Protein intake (g/day)	145	86	84	38	112	71
Fat intake (g/day)	116	58	68	27	90	50

### Procedures

#### Baseline demographics and hemodynamics

During the initial visit, after providing written consent, participants were instructed to rest quietly for approximately 10 min before measuring resting heart rate and blood pressure. Resting heart rate was measured by palpating the radial artery for a period of 60 s. While still resting, blood pressure (Omron BP785, Omron Corporation, Kyoto, Japan) measurements were taken before participants resumed normal activity. Participants then had their body mass determined (Tanita BWB-627A, Tokyo, Japan) and recorded to the nearest ± 0.1 kg upon arrival prior to each study visit. All recorded body masses were compared to ensure the participant was weight stable. Any participant whose body mass changed by more than 2% between consecutive study visits were excluded from participation. Following body mass measurements, height was measured using a standard wall-mounted stadiometer (Tanita, HR-200, Tokyo, Japan) and recorded to the nearest ± 0.5 cm (cm). Fat and fat-free mass was determined using a bioelectrical impedance analyzer (InBody 570, Beverly Hills, California). Participants were required to observe an overnight fast to ensure an accurate determination of body composition. Body composition analysis occurred between 0600 and 1000 h by trained research personnel. All assessments were completed according to device specifications.

#### Dietary monitoring

Prior to their baseline visit, study participants completed a hand-written four-day food record (three week-days and one weekend day). The four-day food log was provided to allow participants to replicate their diets. In addition to the four-day food log, participants were instructed on how to complete the ASA24 online dietary assessment tool (https://epi.grants.cancer.gov/asa24/), for determination of baseline caloric and macronutrient intake. From this information, study participants were asked to replicate their dietary intake prior to each subsequent testing visit. Study participants reported 100% compliance to this protocol.

#### Venous blood collection and processing

Within each supplementation condition, study participants had an indwelling catheter implanted or single stick venipuncture completed using a forearm vein to allow for multiple sample collection. A total of seven venous blood samples (~ 40 mL) were collected (Fig. 2) into ethylenediaminetetraacetic acid (EDTA) Vacutainer tubes for determination of plasma. Two EDTA-coated Vacutainer tubes were used to collect each sample at 0, 30, 60, 90, 120, 180, and 240 min for each of the participant’s study visits. For each collection, tubes were gently inverted 10 times before being centrifuged at 4 °C for 20 min at 2,000 revolutions per minute (rpm) (MegaFuge XFR, Thermo Fisher Scientific, Waltham, MA, USA). After the completion of centrifuging, plasma was aliquoted (~ 600 µL) into separate micro-centrifuge tubes and appropriately labeled with subject identification, condition, and time-point. Once the samples were aliquoted into their respective microcentrifuge tubes, they were stored at − 80 °C for later amino acid analysis.

#### Supplementation protocol

In a randomized, double-blind, crossover fashion, study participants supplemented on a daily basis for two weeks during one study period with a 25-g dose of either milk protein concentrate (= 22.6 g of protein, ULTRANOR™ 9081 milk protein concentrate, Kerry Group PLC., Naas, Ireland) or a 25-g dose of milk protein concentrate with 1 × 10^9^ CFU BC30 (Ganeden BC30, Kerry Inc. Beloit, WI, USA). Each dose was ingested at the same time of day with 237–355 mL of cold tap water. All participants were required to complete a supplementation log to document when each dose of their assigned protein was consumed. During the first assigned study protocol period, participants reported 100% supplement compliance. Upon completion of their first assigned study protocol period, participants observed a three-week washout period by returning to their normal dietary intake and physical activity habits before beginning supplementation for the second study period. At the conclusion of the second study protocol period, participants reported 97% compliance. Study material identity and potency was verified by an independent lab (for probiotic, Q Laboratories, Cincinnati, OH, USA; for protein content and amino acid composition, Table 2, Eurofins, Madison, WI, USA) after the completion of the study. All study materials were blinded by weighing out the required amounts into individual plastic sachets and labeled with non-identifying numbers and letters. All milk protein concentrate was dosed in identical amounts and thus were identical in volume, texture, color, and smell. Maltodextrin and BC30 was added to each sachet and thoroughly mixed which prevented any ability to decipher between two products.

**Table 2:** Composition of ingested proteins (Eurofins, Madison, WI. Report # 2892563-0 and 2892562-0, dated June 3, 2020)

	MPC		MPCBC30	
	AA (mg/serving)	AA (mg/g protein)	AA (mg/serving)	AA (mg/g protein)
Aspartic acid	728	32.2	717	31.8
Threonine	782	34.5	779	34.5
Serine	1590	70.2	1580	70.0
Glutamic acid	157	6.9	155	6.9
Proline	4510	199.2	4510	199.8
Glycine	404	17.8	400	17.7
Alanine	598	26.4	596	26.4
Valine	1120	49.5	1130	50.1
Isoleucine	2130	94.1	2120	93.9
Leucine	1840	81.3	1830	81.1
Tyrosine	613	27.1	611	27.1
Phenylalanine	1060	46.8	1040	46.1
Lysine	2220	98.0	2220	98.4
Histidine	1200	53.0	1200	53.2
Arginine	924	40.8	916	40.6
Cystine	286	12.6	295	13.1
Methionine	1160	51.2	1150	51.0
Tryptophan	1320	58.3	1320	58.5
Total (mg**)**	22,642	1000.0	22,569	1000.0

#### Amino acid determination

Amino acid analysis was performed by Heartland Assays (Iowa State University Research Park, Ames, IA, USA). Plasma samples were assayed for concentration of 20 different amino acids (arginine, glutamine, citrulline, serine, asparagine, glycine, threonine, alanine, ornithine, methionine, proline, lysine, aspartic acid, histidine, valine, glutamic acid, tryptophan, leucine, phenylalanine, isoleucine, cysteine, tyrosine) using a standardized liquid chromatography, mass spectrometry procedure. Briefly, EZ:faast® amino acid analysis kits (Phenomenex, Torrance, CA) were used for liquid chromatographic analysis of amino acids using tandem-mass spectrometry (LC/MS/MS) and electrospray ionization (ESI). The procedure consisted of solid phase extraction of 25 µl of plasma with internal standards by a sorbent tip attached to a syringe with an eluting solvent (a 3:2 mixture of sodium hydroxide with 77% n-propanol, and 23% 3-picoline). The free amino acids were then derivatized by adding a mixture of 17.4% propyl chloroformate, 11% isooctane, and 71.6% chloroform. The resulting mixture was vortexed and allowed to sit at room temperature for 1 min, followed by liquid–liquid extraction with isooctane. 50 µl of the organic layer was removed, dried under nitrogen gas, and suspended in the HPLC run solvents before being injected into the LC/MS/MS. Chromatographic separation of the derivatized amino acids was conducted on an EZ: faast amino acid analysis-mass spectrometry column (250 × 2.0 mm i.d., 4 µm) using a Agilent 6460 triple quadrupole LC/MS/MS system (Santa Clara, CA). 10 mM ammonium formate in water with 0.2% formic acid (mobile phase A) and 10 mM ammonium formate in methanol with 0.2% formic acid (mobile phase B) were used as solvent system with gradient conditions of 68% B at 0 min to 83% B over 13 min with a flow rate of 0.25 ml/min. Amino acids and internal standard data were collected using the Dynamic Multiple Reaction Monitoring mode using Mass Hunter acquisition software (Agilent, Santa Clara, CA). Mass Hunter Quantitation software was used to quantitate the unknown plasma samples based on best fit standard curves.

#### Adverse event reporting

Study participants were asked to verbally report the incidence and severity of any adverse events (dizziness, headache, nausea, upset stomach, cramping, diarrhea, etc.) throughout consumption of either test product.

### Statistical analysis

Primary outcomes for this trial were considered to be the area under the curve (AUC) data for the measured amino acids. Secondary outcomes were considered to the maximum concentrations (C_Max_) identified for the measured amino acids. All analyses were completed using Microsoft Excel and the Statistical Package for the Social Sciences (v23; SPSS Inc., Chicago IL). For all dependent measures, descriptive statistics and presented herein as mean ± standard deviations. Before any statistical tests were completed the normality was assessed for all dependent variables. All non-normal data was log-transformed and then analyzed using both parametric and non-parametric approaches. In all such situations the final statistical decision was identical whether parametric or non-parametric approaches were completed. All reported *p *values are computed using parametric approaches. Paired sample t-tests were completed to determine between-group differences for the AUC, C_Max_, and T_Max_ values for all individually measured amino acids as well as the sum of the branched-chain, essential, and total amino acids. For all statistical tests, data was considered statistically significant when the probability of type I error was 0.05 or less. Between-group effect sizes, *p *values and 95% confidence intervals were computed and are provided in the tables.

## Results

### Amino acids area under the curve (AUC, µmol/L 180 min)

As seen in Table 3, the addition of BC30 led to significantly greater area under the curve values (AUC) for all measured amino acids except for citrulline, asparagine, histidine, glutamic acid, and tryptophan. AUC values for arginine (MPC: 329 ± 84 vs. MPCBC30: 368 ± 66, *p* = 0.028, 11.8% difference, d = 0.52, (95% CI: − 73.2, − 4.5 µmol/L 180 min) and isoleucine (MPC: 384 ± 52 vs. MPCBC30: 403 ± 62, *p* = 0.050, 4.7% difference, *d* = 0.32, (95% CI: − 36.5, 0.00 µmol/L 180 min) were significantly greater in MPCBC30 and tended to be greater for phenylalanine (MPC: 289 ± 59 vs. MPCBC30: 303 ± 41, *p* = 0.072, 4.9% difference, *d* = 0.28, (95% CI: 29.8, 1.4 µmol/L 180 min). Alternatively, glutamic acid tended to be greater in MPC (MPC: 134 ± 40 vs. MPCBC30: 148 ± 53, *p* = 0.088, − 9.4% difference, *d* = − 0.30, (95% CI: − 30.3, 2.2 µmol/L 180 min).

**Table 3:** Individual amino acids, total BCAA, total EAA, and total amino acid area under the curve (AUC, μmol /L 180 min)

Amino acid	MPC		MPC + BC30		MPC vs. MPC + BC30			
	Mean	SD	Mean	SD	*p *value (t-test)	% Difference	ES (d)	95% CI
Arginine	*329*	*84*	*368*	*66*	*0.028*	*11.8*	*0.52*	*(*− *73.2, *− *4.5)*
Glutamine	2,478	281	2,483	307	0.924	0.2	0.02	(− 102.2, 92.9)
Citrulline	148	36	145	28	0.541	− 1.8	− 0.08	(− 6.1, 11.4)
Serine	421	71	439	73	0.131	4.2	0.24	(− 40.7, 5.6)
Asparagine	331	80	312	71	0.261	− 5.8	− 0.26	(− 15.0, 53.3)
Glycine	914	221	967	271	0.095	5.4	0.20	(− 109.0, 9.3)
Threonine	571	124	572	118	0.962	0.2	0.01	(− 47.9, 45.7)
Alanine	1,416	341	1,472	293	0.268	3.9	0.17	(− 156.4, 45.2)
Ornithine	266	61	284	70	0.165	6.9	0.28	(− 44.6, 8.0)
Methionine	131	26	138	19	0.101	6.0	0.34	(− 17.4, 1.6)
Proline	966	213	974	174	0.773	0.8	0.04	(− 63.4, 47.6)
Lysine	826	133	842	117	0.451	2.0	0.13	(− 62.0, 28.3)
Aspartic acid	18	7	19	9	0.824	2.4	0.05	(− 4.8, 3.8)
Histidine	327	44	324	34	0.642	− 0.9	− 0.09	(− 10.1, 16.1)
Valine	1,527	219	1,560	263	0.378	2.1	0.13	(− 107.3, 42.0)
Glutamic acid	134	40	148	53	0.088	9.4	0.30	(− 30.3, 2.2)
Tryptophan	382	74	397	94	0.485	3.8	0.18	(− 58.5, 28.5)
Leucine	770	102	797	122	0.123	3.5	0.24	(− 61.1, 7.7)
Phenylalanine	289	59	303	41	0.072	4.9	0.28	(29.8, 1.4)
Isoleucine	*384*	*52*	*403*	*62*	*0.050*	*4.7*	*0.32*	*(*− *36.5, 0.00)*
Cysteine	72	30	77	34	0.482	7.7	0.17	(− 21.3, 10.3)
Tyrosine	344	59	356	60	0.265	3.6	0.21	(− 34.9, 10.0)
Total BCAA	2,698	364	2,762	433	0.301	2.4	0.16	(− 188.1, 60.2)
Total EAA	4,636	553	4,764	565	0.166	2.8	0.23	(− 313.8, 56.6)
Total Amino Acids	13,048	1,433	13,381	1,140	0.146	2.6	0.26	(− 789.3, 123.0)

Values inside table are the calculated areas under the curves (AUC) using the trapezoidal rule for each participant who completed the study protocol. Italic-type face = between-group difference (p < 0.05)

### Maximum amino acids concentration (C_MAX_, µmol/L)

As seen in Table 4, the addition of BC30 led to significantly greater C_Max_ values for several amino acids. In this respect, C_Max_ values were greater for arginine (MPC: 105 ± 25 vs. MPCBC30: 121 ± 24, *p* = 0.008, 15.0% difference, d = 0.63, (95% CI: − 26.9, − 4.5 µmol/L), serine (MPC: 131 ± 21 vs. MPCBC30: 141 ± 25, *p* = 0.008, 7.8% difference, d = 0.44, (95% CI: − 17.6, − 2.8 µmol/L), glycine (MPC: 261 ± 61 vs. MPCBC30: 277 ± 23, *p* = 0.049, 6.3% difference, d = 0.24, (95% CI: − 32.8, − 0.04 µmol/L), ornithine (MPC: 78 ± 16 vs. MPCBC30: 88 ± 21, *p* = 0.016, 12.6% difference, d = 0.53, (95% CI: − 17.7, − 2.0 µmol/L), methionine (MPC: 42 ± 7 vs. MPCBC30: 45 ± 6, *p* = 0.039, 7.1% difference, d = 0.44, (95% CI: − 5.8, − 0.2 µmol/L), glutamic acid (MPC: 46 ± 16 vs. MPCBC30: 54 ± 22, *p* = 0.021, 17.8% difference, d = 0.43, (95% CI: − 14.9, − 1.3 µmol/L), leucine (MPC: 275 ± 41 vs. MPCBC30: 297 ± 62, *p* = 0.038, 8.1% difference, d = 0.42, (95% CI: − 43.3, − 1.3 µmol/L), phenylalanine (MPC: 89 ± 16 vs. MPCBC30: 95 ± 13, *p* = 0.030, 7.0% difference, d = 0.42, (95% CI: − 11.6, − 0.6 µmol/L), isoleucine (MPC: 142 ± 20 vs. MPCBC30: 154 ± 30, *p* = 0.042, 8.1% difference, d = 0.45, (95% CI: − 22.6, − 0.4 µmol/L), and tyrosine (MPC: 111 ± 18 vs. MPCBC30: 121 ± 25, *p* = 0.024, 8.8% difference, d = 0.44, (95% CI: − 18.4, − 1.4 µmol/L).

**Table 4:** Individual amino acids, total BCAA, total EAA, and total amino acid concentration maximum (C_Max_, µmol/L)

Amino Acid	MPC		MPC + BC30		MPC + BC30 vs. MPC			
	Mean	SD	Mean	SD	*p *value (t-test)	% Difference	ES (d)	95% CI
Arginine	*105*	*25*	*121*	*24*	*0.008*	*15.0*	*0.63*	*(*− *26.9, *− *4.5)*
Glutamine	688	65	701	81	0.373	1.9	0.18	(− 43.0, 16.6)
Citrulline	43	12	44	8	0.833	0.9	0.04	(− 3.5, 2.8)
Serine	*131*	*21*	*141*	*25*	*0.008*	*7.8*	*0.44*	*(*− *17.6, *− *2.8)*
Asparagine	131	21	106	23	0.561	− 2.8	− 0.12	(− 7.4, 13.4)
Glycine	*261*	*61*	*277*	*23*	*0.049*	*6.3*	*0.24*	*(*− *32.8, *− *0.04)*
Threonine	172	33	177	34	0.476	2.7	0.14	(− 17.7, 8.4)
Alanine	416	99	441	92	0.076	6.2	0.27	(− 54.7, 2.9)
Ornithine	*78*	*16*	*88*	*21*	*0.016*	*12.6*	*0.53*	*(*− *17.7, *− *2.0)*
Methionine	*42*	*7*	*45*	*6*	*0.039*	*7.1*	*0.44*	*(*− *5.8, *− *0.2)*
Proline	314	64	331	56	0.163	5.6	0.29	(− 42.7, 7.5)
Lysine	272	40	288	51	0.056	5.9	0.35	(− 32.5, 0.4)
Aspartic acid	6.3	2.3	7.4	5.2	0.302	17.5	0.27	(− 3.3, 1.1)
Histidine	97	12	98	10	0.348	1.9	0.16	(− 5.6, 2.1)
Valine	462	64	493	101	0.057	6.7	0.37	(− 62.9, 1.0)
Glutamic acid	*46*	*16*	*54*	*22*	*0.021*	*17.8*	*0.43*	*(*− *14.9, *− *1.3)*
Tryptophan	122	25	130	33	0.34	6.0	0.25	(− 22.0, 7.3)
Leucine	*275*	*41*	*297*	*62*	*0.038*	*8.1*	*0.42*	*(*− *43.3, *− *1.3)*
Phenylalanine	*89*	*16*	*95*	*13*	*0.030*	*7.0*	*0.42*	*(*− *11.6, *− *0.6)*
Isoleucine	*142*	*20*	*154*	*30*	*0.042*	*8.1*	*0.45*	*(*− *22.6, *− *0.4)*
Cysteine	21	8	23	9	0.372	9.7	0.23	(− 6.4, 2.5)
Tyrosine	*111*	*18*	*121*	*25*	*0.024*	*8.8*	*0.44*	*(*− *18.4, *− *1.4)*
Total BCAA	882	119	942	186	0.055	6.8	0.38	(− 121, 1.3)
Total EAA	*1490*	*170*	*1589*	*248*	*0.020*	*6.6*	*0.47*	*(*− *182, *− *17)*
Total Amino Acids	*3769*	*775*	*4042*	*455*	*0.003*	*6.1*	*0.61*	*(*− *390, *− *90)*

Data provided is means ± SD. Italic-face font = between-group difference (*p* < 0.05). C_Max_ = Maximum observed concentration (in μmol/L) for each condition for each participant who completed study trial

In addition, combining BC30 with MPC also led to significantly greater C_Max_ values for total EAAs (MPC: 1490 ± 170 vs. MPCBC30: 1589 ± 248, *p* = 0.020, 6.6% difference, d = 0.47, (95% CI: − 182, − 17 µmol/L) and total amino acids (MPC: 3769 ± 775 vs. MPCBC30: 4042 ± 455, *p* = 0.003, 6.1% difference, d = 0.61, (95% CI: − 390, − 90 µmol/L) while total BCAAs tended to be greater (MPC:882 ± 119 vs. MPCBC30: 942 ± 186, *p* = 0.055, 6.8% difference, d = 0.38, (95% CI: − 121, 1.3 µmol/L).

### Measured time point for maximum amino acid concentration (T_Max_)

As seen in Table 5, the addition of BC30 led to significantly greater T_Max_ values for glutamine (MPC: 80 ± 57 vs. MPCBC30: 45 ± 22, *p* = 0.002, − 43.8% difference, d = − 0.82, (95% CI: 13.8, 56.2 min), citrulline (MPC: 93 ± 59 vs. MPCBC30: 52 ± 49, *p* = 0.001, − 44.1% difference, d = − 0.76, (95% CI: 17.7, 64.3 min), threonine (MPC: 63 ± 44 vs. MPCBC30: 44 ± 30, *p* = 0.032, − 30.2% difference, d = − 0.55, (95% CI: 1.7, 36.3 min), and alanine (MPC: 78 ± 49 vs. MPCBC30: 54 ± 42, *p* = 0.023, − 30.8% difference, d = − 0.53, (95% CI: 3.5, 44.5 µmol/L). All other measured T_Max_ values were found to not be different conditions.

**Table 5:** Individual amino acids, total BCAA, total EAA, and total amino acid time (minutes) to maximum concentration (T_Max_)

Amino acid	MPC		MPC + BC30		MPC + BC30 vs. MPC			
	Mean	SD	Mean	SD	*p *value (t-test)	% Difference	ES (d)	95% CI
Arginine	46	23	43	20	0.541	− 6.5	− 0.14	(− 6.9, 12.9)
Glutamine	*80*	*57*	*45*	*22*	*0.002*	− *43.8*	− *0.82*	*(13.8, 56.2)*
Citrulline	*93*	*59*	*52*	*49*	*0.001*	− *44.1*	− *0.76*	*(17.7, 64.3)*
Serine	49	27	40	20	0.095	− 18.4	− 0.38	(− 1.7, 19.7)
Asparagine	50	27	45	31	0.524	− 10.0	− 0.17	(− 10.9, 20.9)
Glycine	47	44	39	43	0.451	− 17.0	− 0.18	(− 13.4, 29.4)
Threonine	*63*	*44*	*44*	*30*	*0.032*	− *30.2*	− *0.55*	*(1.7, 36.3)*
Alanine	*78*	*49*	*54*	*42*	*0.023*	− *30.8*	− *0.53*	*(3.5, 44.5)*
Ornithine	72	44	61	29	0.271	− 15.3	− 0.30	(− 9.1, 31.1)
Methionine	49	27	44	25	0.393	− 10.2	− 0.19	(− 6.8, 16.8)
Proline	53	27	49	23	0.502	− 7.5	− 0.16	(− 8.0, 16.0)
Lysine	48	27	44	23	0.502	− 8.3	− 0.16	(− 8.0, 16.0)
Aspartic acid	74	76	73	71	0.954	− 1.4	− 0.01	(− 34.2, 36.2)
Histidine	51	26	42	17	0.107	− 17.6	− 0.41	(− 2.1, 20.1)
Valine	57	26	48	27	0.174	− 15.8	− 0.35	(− 4.2, 22.2)
Glutamic acid	72	69	66	64	0.721	− 8.3	− 0.09	(− 28.0, 40.0)
Tryptophan	56	28	54	27	0.794	− 3.6	− 0.07	(− 13.5, 17.5)
Leucine	46	26	43	26	0.573	− 6.5	− 0.12	(− 7.7, 13.7)
Phenylalanine	48	27	47	26	0.861	− 2.1	− 0.04	(− 10.6, 12.6)
Isoleucine	47	26	43	22	0.459	− 8.5	− 0.17	(− 6.9, 14.9)
Cysteine	49	29	42	41	0.519	− 14.3	− 0.20	(− 15.0, 29.0)
Tyrosine	52	27	46	27	0.297	− 11.5	− 0.22	(− 5.5, 17.5)
Total BCAA	46	26	41	19	0.231	− 10.9	− 0.22	(− 3.4, 13.4)
Total EAA	47	26	45	26	0.712	− 4.3	− 0.08	(− 9.0, 13.0)
Total Amino Acids	48	27	43	22	0.378	− 10.4	− 0.20	(− 6.4, 16.4)

Values inside table are the calculated means ± SD for each respective T_Max_ value. Italic-face font = between-group difference (*p* < 0.05). T_Max_ = Timepoint (in minutes) at which maximum concentration was observed

### Adverse event reporting

MPC and MPCBC30 were very well tolerated. No study participants self-reported any adverse events throughout each study period of the protocol.

## Discussion

A randomized, double-blind, crossover study with an isocaloric and isonitrogenous protein control was completed to investigate the amino acid absorption patterns after consuming daily 25-g doses of either milk protein concentrate or a 25-g dose of milk protein concentrate with BC30 over a two-week period. The primary findings from the study revealed that two amino acids, arginine and isoleucine, exhibited significantly greater areas under the curve when BC30 was added to the milk protein concentrate. Greater peak concentrations of arginine, serine, ornithine, methionine, glutamic acid, phenylalanine, isoleucine, tyrosine, essential amino acids and total amino acids were all found when BC30 was added to milk protein concentrate. Finally, several amino acids (glutamine, citrulline, threonine, and alanine) also exhibited the ability to reach peak concentrations at a faster rate when BC30 was added to milk protein concentrate. Due to the spore-forming ability of BC30, it has been shown to be able to survive the gut and produce certain enzymes (alkaline proteases), which facilitates its ability to heighten protein and carbohydrate digestion [6]. Findings from this study provide further support of BC30′s ability to survive the human gut and support previous in vitro work that document its ability to aid in the breakdown of protein [8, 18]. Notably, not all of the amino acids that were measured experienced increases in the area under the curve outcomes presented herein. In this respect, measured levels of citrulline (− 1.8%), histidine (− 0.9%), and asparagine (− 5.8%) were higher in MPC than MPCBC30. While it was beyond the scope of this investigation to identify mechanistic reasons for the different direction of these outcomes, it remains possible that the amino acid structure prevented appropriate interaction with BC30 or that the associated transporters for these particular amino acids were not as influenced by the presence of BC30. Future research should seek to identify if adding BC30 to other sources of protein may result in similar outcomes. Plant proteins possess varying degrees of amino acid compositions as well as rates of incorporation and digestibility [19]. It is for these reasons that plant proteins and other sources of protein are oftentimes evaluated and compared to each other for their ability to impact health-related outcomes [20]. As amino acid appearance in the blood from plant proteins is lower in comparison to animal proteins, BC30 might even have a greater impact on plant proteins. In addition, results from this investigation will hopefully lead to greater investigations into the potential for the addition of probiotics such as BC30 in other populations, particularly in aging populations whereby protein intakes are known to be reduced and their ability to assimilate protein is underpinned by advancing age [21–23]. In this respect, the addition of BC30 to various protein sources may allow for higher amounts of amino acids to be absorbed into the blood, which can subsequently reduce the protein dose required to be efficacious. This outcome would be particularly attractive for an aging population who typically struggles to consume enough protein both per meal and across an entire-24 h day [21, 22].

A key strength of this investigation centers upon the randomized, double-blind, crossover study design with an isocaloric and isonitrogenous control group using a study sample that was representative of healthy men and women. In addition, a two-week supplementation period was used as part of this study in conjunction with other studies of this nature as this has been shown to be a suitable amount of time for the ingested probiotic to exert physiological outcomes [7, 24]. Compliance to the supplementation protocol was high (> 90%) and all collected blood samples were processed and analyzed under identical conditions. Our study was limited by our inability to collect urine and fecal samples that would have allowed for better understanding of how much protein is being excreted and incorporated into body tissues. In this respect, amino acid concentrations in the blood do not directly relate to muscle or whole-body protein synthesis and follow-up work that involves these analyses and the collection of skeletal muscle biopsy samples would be needed.

## Conclusion

In conclusion, results from the present study indicate that coadministration of BC30 over a two-week period to 25-g doses of milk protein increases the post-prandial changes in area under the curve, concentration max, and time to reach concentration max in healthy men and women. These results provide additional evidence that adding probiotics to various forms of protein can improve the appearance of amino acids in the blood. This outcome may have particular importance for those people who would prefer to take a smaller dose of protein or due to factors such as aging or gastrointestinal compromise may lack the digestive efficiency required to assimilate larger doses of protein.

## Data Availability

The datasets used and/or analyzed during the current study are available from the corresponding author upon reasonable request.

## Abbreviations

yr: Year
cm: Centimeter
kg: Kilogram
g: Gram
mL: Milliliters
L: Liter
BIA: Bioelectrical impedance analysis
ASA24: Automated self-administered 24-hour dietary assessment tool
^o^C: Degrees celsius
rpm: Revolutions per minute
BCAA: Branched chain amino acids
EAA: Essential amino acids
AA: Amino acids
AUC: Area under the curve
C_Max_: Maximum amino acid concentration
T_Max_: Measured time point for maximum amino acid concentration
µmol-h/L: Millimoles an hour per liter
µmol/L: Millimoles per liter
CONSORT: Consolidated Standards of Reporting Trials
